# Effects of Trans, Trans-2,4-decadienal on the Ions Currents of Cardiomyocytes: Possible Mechanisms of Arrhythmogenesis Induced by Cooking-oil Fumes

**DOI:** 10.1038/s41598-020-62733-1

**Published:** 2020-04-01

**Authors:** Shih-Jie Jhuo, I-Hsin Liu, Wei-Chung Tsai, Kun-Tai Lee, Bin-Nan Wu, Wen-Ter Lai

**Affiliations:** 1 Division of Cardiology, Department of Internal Medicine, Kaohsiung Medical University Hospital, Kaohsiung, Taiwan; 2 Graduate Institute of Clinical Medicine, Kaohsiung Medical University, Kaohsiung, Taiwan; 3 Department of Internal Medicine, Faculty of Medicine, College of Medicine, Kaohsiung Medical University, Kaohsiung, Taiwan

**Keywords:** Ion transport, Arrhythmias

## Abstract

Household air pollution has adverse effects on cardiovascular health. One of the major sources of household air pollutants is the combustion of cooking oils during cooking. Trans, trans-2,4-decadienal (tt-DDE) is a type of dienaldehyde that is present in a wide range of food and food products. It is a byproduct of the peroxidation of linoleic acid following the heating of oil during cooking. The mechanisms of the associations between household air pollution and cardiac arrhythmias are currently unclear. The purpose of this study was to determine effects of tt-DDE on the ion currents in H9c2 cells. The I_K_ and I_Ca,L_ in H9c2 cells treated with and without tt-DDE were measured using the whole-cell patch clamp method. Expressions of Kv2.1 and Cav1.2 in H9c2 cells treated with and without tt-DDE were measured by western blot analysis. After the H9c2 cells had been exposed to tt-DDE, the I_K_ and I_Ca,L_ were significantly decreased. The expression of Kv2.1, unlike that of Cav1.2, was also significantly decreased in these cells. These changes in I_K_ and I_Ca,L_ that were induced by tt-DDE may help to explain the association between cardiac arrhythmogenesis and cooking-oil fumes.

## Introduction

Epidemiological studies have revealed that exposure to air pollutants, especially particulate matter (PM), is associated with an increased incidence of respiratory diseases, as well as of cardiovascular morbidity and mortality^1,2^. Exposure to air pollution could increase the risks of ischemic heart disease, heart failure, cerebrovascular disease, and cardiac arrhythmias^3,4^. In the Air Pollution and Cardiac Risk and its Time Course (APACR) study, 60 minutes of exposure to PM_2.5_ (particles with a diameter of ≤2.5 μm) was associated with increased premature ventricular contractures^5^. Another study demonstrated that brief exposure to air pollution could trigger atrial fibrillation^6^. The mechanisms of the associations between air pollution and cardiovascular diseases are currently unclear. It has been proposed that increased systemic inflammatory responses, systemic oxidative stress, systemic and pulmonary artery blood pressure, and risks of atherosclerosis, as well as changes in autonomic function indicate increased cardiovascular morbidity and mortality caused by air pollution^7–9^.

As comprehensively demonstrated in previous studies, household air pollution also has adverse effects on cardiovascular health^10^. Indoor exposures to PM_2.5_ is associated with alternation of heart rate variability that could in turn increase vulnerability to cardiac arrhythmias^11^. One of the major sources of household air pollutants is the combustion of cooking oils during cooking. A longitudinal study conducted by Chinese military cooks showed that exposure to compounds derived from cooking-oil fumes could cause oxidative DNA damage and lipid peroxidation^12^. However, the effects of the contents of cooking-oil fumes on vulnerability to cardiac arrhythmias is unknown. Trans, trans-2,4-decadienal (tt-DDE) is a type of dienaldehyde that is present in a wide range of food and food products. It is a byproduct of the peroxidation of linoleic acid following the heating of oil during cooking^13^. The alteration of ions currents in cardiomyocytes is one of the mechanisms involved in arrhythmogenesis. The purpose of this study was to determine these effects of tt-DDE on the ions currents in cardiomyocytes.

## Results

### Cytotoxicity of tt-DDE

**Figure 1:**
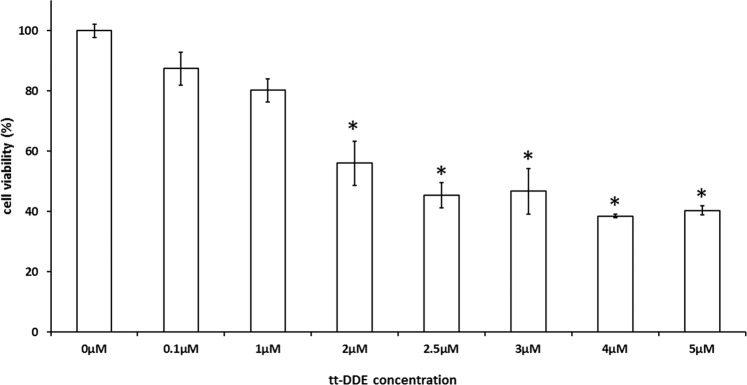
Viability of H9c2 cells after they had been co-cultured with different concentrations of tt-DDE for 24 h. After 24 h, tt-DDE at concentrations of ≥2 µM led to a significant reduction of cell viability. Data are mean ± standard deviation. Each measurement was performed in triplicate *P < 0.05 compared with 1 µM.

Figure 1 demonstrated viability of H9c2 cells after they had been co-cultured with different concentrations of tt-DDE for 24 hours. After the treatments of tt-DDE with concentrations of 0.1 µM and 1 µM, respectively, the H9c2 cellular viability was not significantly different (87.50 ± 5.44% v.s. 80.2 ± 3.85%; P = 0.14). The treatments with 1 µM and 2 µM of tt-DDE, respectively, for 24 hours resulted in a markable decrease in the viability of H9c2 cells, from 80.2 ± 3.85% to 56.07 ± 7.38% (P < 0.05) and tt-DDE at concentrations of ≥2 µM led to a significant reduction of cell viability compared with at a concentration of 1 µM (P < 0.05). To determine the effects of tt-DDE on delayed-rectifier potassium outward current (I_K_) and L-type calcium channel current (I_Ca,L_), H9c2 cells were co-cultured with tt-DDE at a concentration of 1 µM.

### I_K_ in H9c2 cells treated with tt-DDE-free media

**Figure 2:**
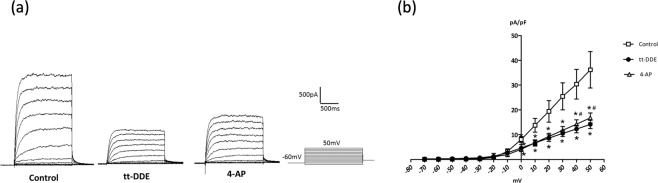
(**a**) Representative current traces for the delayed rectifier K^+^ outward currents (I_K_) measured in H9c2 cells after treatment with tt-DDE-free media for 18 h (control group), tt-DDE-containing media for 18 h (1 µM; tt-DDE group) and 4-amiopyridine (5 mM; 4-AP).The I_K_ were elicited by 300 ms of depolarizing step pulses from −70 to +50 mV at a holding potential of −60 mV. The I_K_ were suppressed significantly by 4-AP. (**b**) The average relationship between the I_K_ (pA/pF) and membrane potential in the control group (squares), the tt-DDE group (black circles) and 4-AP group (triangles) (n = 10). Compared with the control group, the I_K_ were significantly decreased at membrane potentials from 0 to +50 mV in the tt-DDE group and 4-AP groups. Compared with 4-AP group, the I_K_ were significantly decreased at membrane potentials from 40 to 50 mV in the tt-DDE group. *P < 0.05 compared with the control group. ^#^P < 0.05 compared with the 4-AP group.

I_K_ were evoked in the H9c2 cells treated with tt-DDE-free media for 18 hours (control group) with 300 ms of depolarizing step pulses from −70 to +50 mV in 10 mV increments at a holding potential of −60 mV. As described in a previous report^14^, I_K_ first appeared significantly at the membrane potential of −20 mV and the currents amplitude increased with more positive membrane potentials until it reached the at membrane potentials of 50 mV (Fig. 2a). The I_K_ was suppressed significantly by 4-amiopyridine (4-AP) (Fig. 2a).

### Effects of tt-DDE on I_K_

After treatment with tt-DDE-containing media for 18 hours (tt-DDE group), I_K_ were evoked in the H9c2 cells with 300 ms of depolarizing step pulses from −70 to +50 mV in 10 mV increments at a holding potential of −60 mV. Compared with the activation pattern of the I_K_ in the control group, the I_K_ amplitudes in the cells of this group were significantly smaller (Fig. 2a). The average relationships between the I_K_ (pA/pF) currents and membrane potentials in the control and tt-DDE groups are analyzed in Fig. 2b (n = 10), which shows that the I_K_ in the tt-DDE group were significantly lower than those in the control group from 0 to 50 mV (all P < 0.05; Fig. 2b).

### I_Ca,L_ in H9c2 cells treated with tt-DDE-free media

**Figure 3:**
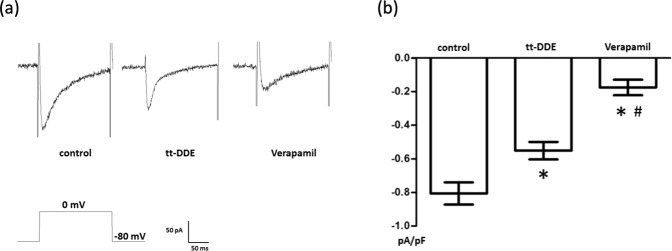
(**a**) Representative current traces for the L-type Ca^2+^ channel current (I_Ca,L_) measured in H9c2 cells after treatment with tt-DDE-free media for 18 hours (control group), tt-DDE-containing media for 18 hours (1 µM; tt-DDE group) and verapamil (1 μM). The I_Ca,L_ were elicited by a depolarizing single pulse of 0 mV for 300 ms at a holding potential of −80 mV. The I_Ca,L_ were significantly suppressed by verapamil. (**b**) Comparison of the I_Ca,L_ density in the control, tt-DDE and verapamil groups. The I_Ca,L_ were significantly decreased in the tt-DDE and verapamil groups than in the control group (n = 6). Data are mean ± standard deviation; *P < 0.05 compared with the control; ^#^P < 0.05 compared with the tt-DDE group.

A depolarizing single pulse with 0 mV was applied for 300 ms at a holding potential of −80 mV to record the inward I_Ca,L_ current in the control group. The I_Ca,L_ current was recorded, and it was significantly suppressed by verapamil (Fig. 3a).

### Effects of tt-DDE on the I_Ca,L_

I_Ca,L_ in the tt-DDE group were evoked by a depolarizing single pulse with 0 mV for 300 ms at a holding potential of −80 mV (Fig. 3a). The I_Ca,L_ current were significantly decreased in this group than in the control group (−0.55 ± 0.05 v.s. −0.81 ± 0.07 pA/pF, respectively, n = 6; P < 0.001; Fig. 3b).

### Effects of tt-DDE on the expression of Kv2.1 and Cav1.2

**Figure 4:**
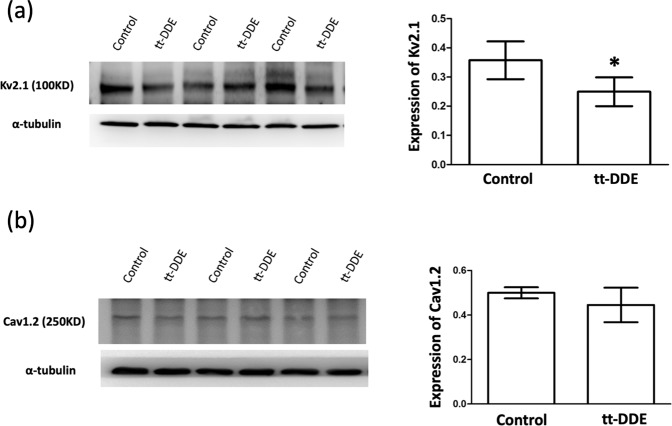
Expression of Kv2.1 (**a**) and Cav1.2 (**b**) measured by western blotting in the H9c2 cells after treatment with tt-DDE-free media for 18 hours (control group), tt-DDE-containing media for 18 hours (1 µM; tt-DDE group). Compared with the control group, the expression of Kv2.1 was significantly decreased in the tt-DDE group. In contrast, expression of Cav1.2 did not significantly differ between the two groups. Data are mean ± standard deviation; *P < 0.05 compared with the control.

The I_K_ and I_Ca,L_ recorded in the H9c2 cells were generated by the activation of potassium (K^+^) channels and L-type calcium (Ca^2+^) channels composed mainly of Kv2.1 α-subunits and Cav1.2 subunits, respectively^14,15^. Compared with the control group, the expressions of Kv2.1 in the tt-DDE group was significantly decreased (0.36 ± 0.06 v.s. 0.25 ± 0.05; P = 0.003) (Fig. 4a). The expression of Cav1.2 in the control and tt-DDE groups was 0.5 ± 0.02 and 0.44 ± 0.07, respectively, which was not a statistically significant difference (P = 0.35) (Fig. 4b).

## Discussions

After the H9c2 cells had been exposed to tt-DDE, the I_K_ and I_Ca,L_ were significantly decreased. The expression of Kv2.1, a component of the K^+^ channel, unlike that of Cav1.2, a component of the Ca^**2+**^ channel, was also significantly decreased in these cells. These changes in I_K_ and I_Ca,L_ that were induced by tt-DDE may help to explain the association between cardiac arrhythmogenesis and cooking-oil fumes.

Many studies have demonstrated an association between air pollution and cardiac arrhythmias^4–7^. Gold *et al*. investigated the association between the level of ambient pollution and cardiovascular function^3^. They found that exposure to PM_2.5_ and ozone could decrease vagal tone, resulting in reduced heart rate variability. As previous studies, air pollution is associated with changes in cardiac ventricular repolarization, regardless of the duration of exposure^7,16^. The APACR study showed that increases of 10 µg/m^3^ in the average PM_2.5_ concentration were associated with 8% and 3% increases in the average premature ventricular contraction count in the same and subsequent 30 min periods, respectively^5^. Link *et al*. studied the association between air pollution and cardiac arrhythmias in patients with implantable cardioverter defibrillators. They found that PM_2.5_ exposure was associated with an increased risk of atrial fibrillation within the subsequent hours in patients with known cardiac disease^6^. Indoor air pollution has also contributed to changes in heart rate variability and electrocardiography. A study of housewives in Taiwan showed that indoor exposure to PM_2.5_, especially during stir-frying, cleaning with detergents, and burning incense, was associated with changes in the heart rate variability index^11^. Collectively, these results suggest that indoor air pollution, especially in the kitchen, may contribute to cardiac arrhythmogenesis. However, the mechanisms by which arrhythmogenesis is caused by air pollution have not been well studied.

Cooking-oil fumes are major air pollutants in the kitchen. There is abundant tt-DDE in cooking-oil fumes, and exposure to cooking-oil fumes containing tt-DDE could induce oxidative stress and genotoxicity in human lung cells^13^. Also, tt-DDE could induce the generation of reactive oxygen species and cell death in lung cells^17^. However, the cytotoxicity of tt-DDE to cardiomyocytes is seldom discussed.

Triggered activity, abnormal automaticity, and re-entry are major mechanisms of cardiac arrhythmogenesis. Alterations of function in ion channels and ion currents in cardiomyocytes could result in triggered activity and cardiac arrhythmias. In the current study, we found that after 18 hours of exposure to tt-DDE, the I_K_ and I_Ca,L_ in H9c2 cells were significantly decreased. This effect may prolong the duration of the action potential of cardiomyocytes, which could increase vulnerability to cardiac arrhythmogenesis.

This study also showed that the expression of Kv2.1 was significantly decreased in H9c2 cells treated with tt-DDE. This decrease in the expression of Kv2.1 in H9c2 cells could be one of the mechanisms of decreasing the I_K_ in H9c2 cells treated with tt-DDE. In contrast, the expression of Cav1.2 in the H9c2 cells was not significantly affected by treatment with tt-DDE. This suggests that the toxic effect of tt-DDE on the I_Ca,L_ in cardiomyocytes may not directly affect the L-type Ca^**2+**^ channel. The effects of tt-DDE on the I_K_ and I_Ca,L_ in cardiomyocytes should be further investigated.

### Study limitations

There are some study limitations in this study. First, the effects of tt-DDE on the I_K_ and I_Ca,L_ of H9c2 cells were determined using only one concentration of tt-DDE (1 µM). We did not analyze effects of tt-DDE on I_K_ and I_Ca,L_ with different concentrations of tt-DDE. Results of this study can not conclude the concentration-related effects of tt-DDE on the I_k_ and I_Ca,L_ of H9c2 cells. However, results of this study suggested that tt-DDE could affect the I_k_ and I_Ca,L_ of H9c2 cells. Second, although decrease of the I_K_ and I_Ca,,L_ of H9c2 cells by exposure to 1 µM tt-DDE demonstrated *in vitro* in this study, how much and how long exposure to cooking-oil fume could achieve similar effects by exposure of 1 µM tt-DDE *in vivo* were unknown. Further studies *in vivo* are needed.

## Conclusion

Exposure to tt-DDE has adverse effects leading to decrease in the I_K_ and I_Ca,L_ of cardiomyocytes. This decrease in the I_K_ may be related to decreased expression of the Kv2.1. Decreases in both I_K_ and I_Ca,L_ of cardiomyocytes could prolong the action potential of cardiomyocytes and contribute toward vulnerability to arrhythmogenesis. These may be the mechanisms involved in the development of cardiac arrhythmias associated with exposure to cooking-oil fumes.

## Methods

### Cell culture

We used H9c2 cells (ATCC CLR-1446; Rockville, MD, USA) derived from rat embryonic myoblasts as an *in vitro* model of cardiomyocytes biology, since they show similar ions currents responses to primary adult and neonatal cardiomyocytes. The cells were plated onto collagen-coated culture dishes and cultured in Dulbecco’s Modified Eagle’s Medium supplemented with 10% fetal bovine serum in a humidified atmosphere of 5% CO_2_ and 95% air at 37 °C^14,18^. Cells were used under the 20th passage.

### Cytotoxicity assay

The viability of H9c2 cells in tt-DDE-containing media was determined in an assay using the Cell Counting Kit-8 (CCK-8; Dojinodo Molecular Technologies, Gaithersburg, MD, USA). The CCK-8 assay was based on the conversion of a water-soluble tetrazolium salt, 2-(2-methoxy-4-nitrophenyl)-3-(4-nitrophenyl)-5-(2,4-disulfophenyl)-2H-tetrazolium, monosodium salt, to a water-soluble formazan dye upon reduction by dehydrogenases in the presence of an electron carrier^19^. The CCK-8 assay was performed according to the kit’s manual protocol. Briefly, the H9c2 cells were seeded in 96-well plates and incubated either with 0.1% DMSO or one of several concentrations (0.1 µM, 1 µM, 2 µM, 2.5 µM, 3 µM, 4 µM, and 5 µM) of tt-DDE for 24 hours. The CCK-8 solution was added before the cells were incubated for 3 hours at 37 °C. The absorbance of each well was measured at 450 nm using a Microplate Reader (Bio-Rad, Hercules, CA, USA). Each measurement was performed in triplicate.

### Determination of I_K_ and I_Ca,L_ in H9c2 cells

The I_K_ were measured using the whole-cell patch clamp method previously described in detail^14,20^. In brief, H9c2 cells treated with (tt-DDE group) or without (control group) tt-DDE-containing media for 18 hours were detached with 0.25% trypsin-0.02% EDTA solution, the supernatant was removed by centrifugation, and the pellets were resuspended in 1 ml of bath solution containing (in mM): 60 NaCl, 80 Na-gluconate, 0.1 CaCl_2_, 1 MgCl_2_, 5 KCl, 10 HEPES, and 10 glucose (pH 7.4, NaOH). A recording electrode was pulled from borosilicate glass (resistance: 4–7 MΩ), and the pipette was coated with sticky wax close to the tip to reduce capacitance, backfilled with pipette solution containing (in mM): 0.5 MgCl_2_, 30 KCl, 110 K-gluconate, 10 EGTA, 5 HEPES, 5 Na_2_ATP, and 1 GTP-tris (pH 7.2, KOH). and gently lowered onto an H9c2 cell. Negative pressure was briefly applied to rupture the membrane, and a gigaohm seal was obtained. Cells were subsequently voltage clamped. The I_K_ were recorded using an Axopatch 700 A amplifier (Axon Instruments, Union City, CA, USA), filtered at 1 kHz using a low-pass Bessel filter, digitized at 5 kHz, and stored on a computer for subsequent analysis with Clampfit 10.2(Molecular Devices, San Jose, CA, USA). A 1 M NaCl-agar salt bridge between the bath and the Ag-AgCl reference electrode was used to minimize offset potentials. All electrical recordings were performed at room temperature.

To measure the I_Ca,L_ through L-type Ca^2+^ channels, perforated whole-cell patch clamp electrophysiology was used in H9c2 cells treated with (tt-DDE group) or without (control group) tt-DDE-containing media (1 µM) for 18 hours. This technique was previously described in detail^18^. In brief, H9c2 cells were placed in a recording dish and perfused with a bath solution containing (in mM): 135 tetraethylammonium (TEA)-Cl, 1.8 CaCl_2_, 2 MgCl_2_, 10 glucose, and 10 HEPES (pH 7.4, Tris) using a fire polished glass pipette. To minimize outward potassium currents, Cs^+^ rather than K^+^ was used in the pipette solution. A recording electrode was pulled from borosilicate glass (resistance: 3–5 MΩ), and the pipette was coated with sticky wax close to the tip to reduce capacitance and backfilled with pipette solution containing (in mM): 140 CsCl, 1 EGTA, 1 MgCl_2_, 5 Na_2_ATP, and 5 HEPES (pH 7.2, Tris). Membrane currents were recorded using the MultiClamp 700 A amplifier, filtered at 1 kHz using a low-pass Bessel filter, digitized at 5 kHz and stored on a computer for subsequent analysis with Clampfit 10.2. A 1 M NaCl-agar salt bridge between the bath and the Ag-AgCl reference electrode was used to minimize offset potentials. All electrical recordings were performed at room temperature.

### Western blot analysis

The protein levels of Kv2.1 and Cav1.2 in the H9c2 cells were analyzed using western blotting. The methods used in western blotting have previously been described in detail^14,18^. In brief, total protein content was extracted using a Bio-Rad Protein Assay (Bio-Rad Laboratories, Hercules, USA) and subsequently separated using a 10% denaturing acrylamide gel. The proteins were transferred to Immobilon PVDF membranes (Merck Millipore, Darmstadt, Germany) and incubated with rabbit polyclonal antibodies for Kv2.1 (Merck Millipore, Darmstadt, Germany) and Cav1.2 (Sigma-Aldrich, Darmstadt, Germany) for 1 hour at room temperature. Antibodies were diluted in TBS-Tween 1:500 for anti-Kv2.1 and anti-Cav1.2. Membranes were incubated with a secondary antibody (Merck Millipore, Darmstadt, Germany) conjugated with horseradish peroxidase. Antigen-antibody complexes were detected using enhanced chemiluminescence (Thermo Fisher Scientific Inc., Rockford, USA). Densitometric analysis was conducted using LabWorks 4.5 ImageAcquisition and Analysis software (Ultra-Violet Products Ltd., Cambridge, UK). Each measurement was performed in triplicate.

### Statistical analysis

All data were expressed as mean ± standard deviation, with n indicating the number of cells. Continuous variables in the two groups (control and tt-DDE groups) were compared using non-parametric tests for two independent samples (Mann-Whitney *U* test). The P value less than 0.05 was considered significant. All statistical analyses were performed using SPSS software (version 11.0, SPSS, Chicago, IL, USA).
